# That’s not what you expect to do as a doctor, you know, you don’t expect your patients to die.” Death as a learning experience for undergraduate medical students

**DOI:** 10.1186/s12909-016-0631-3

**Published:** 2016-04-14

**Authors:** Kelby Smith-Han, Helen Martyn, Anthony Barrett, Helen Nicholson

**Affiliations:** Department of Anatomy, Otago School of Medical Sciences, University of Otago, PO Box 56, Dunedin, 9054 New Zealand; Lady Cilento Children’s Hospital, 501 Stanley street, Southbank, Brisbane, 4101 Qld Australia; Otago Medical School, University of Otago, PO Box 56, Dunedin, 9054 New Zealand; University of Otago, PO Box 56, Dunedin, 9054 New Zealand

**Keywords:** Death and dying, Medical students, Undergraduate, End-of-life care, Hidden curriculum, Social organizations, Clinical settings

## Abstract

**Background:**

Experiencing the death of a patient can be one of the most challenging aspects of clinical medicine for medical students. Exploring what students' learn from this difficult experience may contribute to our understanding of how medical students become doctors, and provide insights into the role a medical school may play in this development. This research examined medical students' responses of being involved personally in the death of a patient.

**Method:**

Ten undergraduate medical students were followed through their three years of clinical medical education. A total of 53 individual semi-structured interviews were conducted. Grounded theory analysis was used to analyze the data.

**Results:**

Students illustrated a variety of experiences from the death of a patient. Three main themes from the analysis were derived: (i) Students’ reactions to death and their means of coping. Experiencing the death of a patient led to students feeling emotionally diminished, a decrease in empathy to cope with the emotional pain and seeking encouragement through the comfort of colleagues; (ii) Changing perceptions about the role of the doctor, the practice of medicine, and personal identity. This involved a change in students’ perceptions from an heroic curing view of the doctor’s role to a role of caring, shaped their view of death as a part of life rather than something traumatic, and resulted in them perceiving a change in identity including dampening their emotions; (iii) Professional environment, roles and responsibilities. Students began to experience the professional environment of the hospital by witnessing the ordinariness of death, understanding their role in formalizing the death of a patient, and beginning to feel responsible for patients.

**Conclusions:**

Along with an integrative approach to facilitate students learning about death, we propose staff development targeting a working knowledge of the hidden curriculum. Knowledge of the hidden curriculum, along with the role staff play in exercising this influence, is vital in order to facilitate translating the distressing experiences students face into worthwhile learning experiences. Finally, we argue that student learning about death needs to include learning about the social organization and working life of clinical settings, an area currently omitted from many medical education curricula.

## Background

One of the most challenging aspects of learning about clinical medicine is experiencing the death of a patient. Considering the emotional distress that a death of a patient can have among medical students [1] it is surprising that there has only been a limited amount of literature published concerning medical students’ *personal* experience of the death of a patient. By ‘personal’ we mean that students interacted directly with the patient and had some involvement in their care and/or learning about them as a person.

Previous research has involved students reflections of experiencing the death of a patient before it happens [2, 3], focusing on how to prepare students from a pedagogical or curriculum perspective [4, 5], or looking at students’ accounts of death in general, yet not with patients with whom they have been directly involved [6].

A handful of studies exploring medical students personal experiences of death have only recently appeared [7–11]. All of these studies had the primary aim of examining medical students’ experiences of the death of a patient. The aforementioned research findings included significant emotional responses from students, varying coping strategies undertaken, support from clinicians (in a positive manner or being non-existent), the meaning of a patient death, and the juxtaposition of portraying empathy and yet becoming emotionally detached. One aspect of note is that the research looking into medical students’ personal experiences of death is with medical students who attend graduate medical schools, where students were older than the majority of those in an undergraduate program.

This research was conducted as part of a longitudinal study following undergraduate medical students in New Zealand from Otago Medical School through their entire medical degree and into their postgraduate training. The primary aim of the study is to explore how medical students’ change in their attitude towards medicine, their professional and emotional development, and what factors are involved in their choice of specialty.

This study relates to the primary aim of exploring medical students professional and emotional development, with a supplementary aim to explore how medical students experienced the death of a patient during their clinical years attending a medical program. In particular, we were interested in any change in students’ perceptions during their experiences of a death of a patient.

## Method

### Participants

Initially all students in the first year of the medical program at the Otago Medical School, University of Otago, were invited to participate in the research. A total of 16 students were purposively selected to reflect the make-up of the student cohort (gender, age, ethnicity, entry pathway into medicine). Of the 16 who were invited to participate, ten students were available to be interviewed throughout the clinical years of the program.

The medical program at Otago involves two years pre-clinical education (Early Learning in Medicine, ELM) and three years clinical education (Advanced Learning in Medicine, ALM, years). Entry into the medical program is through a Health Sciences First Year course (~70 % of the total medical school cohort), graduate entry (~20 % of the cohort) and the alternative category (greater than three years since graduating from a previous degree ~ 10 % of the cohort).

Ethical approval was granted for this study from the New Zealand Multi-region Ethics Committee. Informed consent for involvement in the study was obtained from the participants.

### Interviews and analysis

Individual semi structured interviews were conducted. The interviews combined a conversational strategy with an interview guide approach [12]. Examples of topic areas of questions included how students reacted to sickness, suffering and death and what they learned about themselves, others and about the human condition. The same interviewer was used for all interviews (PT – see Acknowledgements). The interviewer was medically qualified but was not involved in any teaching at the medical school or clinical teaching sites.

Students were interviewed throughout each clinical year. This was to capture the various experiences that can occur at any moment in the students’ education such as experiencing the death of a patient. The number of times a student was interviewed was not pre-determined, as this was due to both student and interviewer availability.

Due to the serendipitous nature of participants being personally involved with the death of a patient, it occurred to participants only once in their clinical years, a non-sequential analysis was used rather than the interviews being analysed in a series. Interviews were transcribed and a thematic analysis was conducted using grounded theory analysis [13]. Participants reviewed their interview to check for accuracy as a means of internal validity. The software program ATLAS.ti (ATLAS.ti GmbH, Berlin, Germany) was used for the qualitative analysis [14]. An inductive open coding process was used and emergent themes were generated from an iterative approach of multiple passes over the data. Independent parallel coding and theme generation was initially developed by HM (a final year medical student) and AB (Medical Education Advisor and Researcher) as the second coder. Axial coding was then performed, relating concepts to each other and grouping the codes into larger themes. This was performed via the memo function in ATLAS.ti by KSH (Postdoctoral Fellow in Medical Education). HN (Professor of Anatomy), AB and KSH discussed and negotiated the themes that were emerging during this process until reaching agreement.

The constant comparison method was continually used comparing themes identified and themes beginning to emerge looking for similarities and differences. HN and KSH checked the clarity of the themes with all of the transcripts. Disagreement about the concept and/or assignment of themes led to a further discussion until concordance was achieved.

The continuous revisions of the themes were then refined by AB, HN, and KSH into the final three main themes illustrated in the results. It is important to note that the results are based on changes reported by individual students over time and that the term ‘change’ refers to a self-reflective acknowledgement that students had changed in their perceptions.

### Findings

There were 4 male and 6 female students. Six students were aged 24 years or younger with four aged between 27–30 years (as of the first clinical year) and the group represented all entry pathways into the program.

For this study a total of 53 interviews were completed varying in length from 20 to 30 min with the cohort of ten students. The interviews were completed during students’ time on the hospital wards and general practice placements when they had time available. The number of interviews varied per student in each clinical year due to the availability of the student and interviewer. Six students were interviewed twice in each clinical year, three students being interviewed twice in their first and second clinical years and once in their final year, and one student being interviewed once in each of their clinical years.

One significant influence of the availability of student and interviewer was due to different geographical locations. Otago Medical School operates at three main sites in New Zealand: Dunedin, Christchurch and Wellington. However, due to the sensitive nature of the topics being discussed (e.g. death and dying), maintaining one-one interviews in person was considered to be more appropriate to maintain quality, rather than considering alternative methods to increase availability and frequency of interviews (e.g. Skype or telephone).

All ten students had experienced the death of a patient in their clinical years. The circumstances of the deaths ranged from acute myocardial infarction, expected death of terminally ill patients, the acute unexpected death of a baby and that of a young fit sportsman. The settings where the students experienced the patient deaths were varied, from different wards in a hospital environment, to a placement at a hospice. The deaths affected the students in different ways and the students’ reactions and attitudes towards death changed during their clinical education.

The findings from the analysis identified three major themes that are described below. Pseudonyms are used for confidentiality.

### Students’ reactions to death and methods of coping

For one of our students the first death they experienced was a young fit male only one year older than herself. It had a profound impact on her and led her to become emotionally blunted or to “switch off” her emotions. The student was unaware of how much this experience affected her.

“Straight afterwards it was just a shock, like I cried and did all the normal shock stuff, and I thought about it quite a lot, but then I was surprised by how quickly I thought I got over it but how quickly I stopped thinking about it, you know, by how quickly it didn’t bother me anymore, and I thought, I thought that that was that and I dealt with it, and it wasn’t til a few weeks, oh maybe even a couple of months later that I realized actually it had affected me more than I’d thought, in the sense that now, I just didn’t care about anything.” (Jemma, 1^st^ year of clinical medicine)

The dilemma faced by many students was the balancing act of being able to protect themselves by becoming emotionally detached, yet still being able to display empathy and caring towards their patients.

“That’s almost the trick of medicine, how can you feel enough to show empathy and understand what people are going through and have people appreciate that you do actually, you are actually concerned with what’s going on but not take it all on yourself so that every time you see something tragic or every time you see something horrible, you break down and can’t do anything.” (Kirsten, final year of clinical medicine)

Over time students developed skills and strategies for coping with death and difficult situations. These involved discussing cases with colleagues and friends, or having something to escape to such as exercise, a hobby, or a cold beer:

“I guess in some ways I’ve learnt to develop better coping strategies and those coping strategies surprisingly do evolve around family, friends and beer. Whether I like to admit it or not, after a hard day I like to find one of my colleagues or one of my friends and go out for a beer, just the one beer, to unwind and to just let out any frustration and then get on with it.” (Kate, 2^nd^ year of clinical medicine)

Our students acknowledged the role of supportive staff and a supportive work environment when confronted by death.

“If I hadn’t have had support straight afterwards, sort of explaining what had happened, oh there’s a good chance I wouldn’t have turned up the next day or the day after that, or never again because that’s not what you expect to do as a doctor, you know, you don’t expect your patients to die, it’s not a good introduction to medicine I don’t think.” (Michael, final year of clinical medicine)

### Changing perception of the role of the doctor, the practice of medicine, and personal identity

Through having frequent experiences with death in the clinical years, students began to alter their perceptions of the role of the doctor. One student commented on how their perception of the doctor’s role as a hero and as a curative role “playing God” and “saving lives”, changed to a caring view through management of the illness.

“We can’t play God, we can’t save everybody, you can’t change the outcome for every person, people are inevitably going to die, things are inevitably going to go wrong and you can’t stop that, the best you can do is minimize the risk and…that’s changed my attitude in that I’m probably now less naive about what I expect to happen.” (John, 1^st^ year of clinical medicine)

The experience of spending time at a hospice changed a students’ view of the role of medicine, from trying to ‘cheat death’ to preserving the quality of life and helping patients transition to a more dignified and less anxious death. The experience also shaped their view of death as a part of life, and not a ‘horrible monster’ as first thought:

“Death in the hospital is a terrible thing, oh my God, because of course in medicine, the aim of the game is to cheat death, mainly at least that’s what I always thought, and here [the hospice] the aim of the game is to not cheat death, because we can’t cheat death; it’s to facilitate a comfortable transition from life into death. I mean I’ve already come to terms with death but this just further shows me that it’s not this horrible monster that I always thought it was, it’s…it’s just part of life and it can be made easier and less daunting.” (Theo, final year of clinical medicine)

Students acknowledged that their experiences led, overtime, to viewing death as less traumatic.

“So I guess in some ways going through med school does make you a bit jaded, you do see death more often as you go through and it stops being quite as traumatic but I guess at the same time you have a better understanding of why things happened so you can rationalize it for yourself” (Miles, final year of clinical medicine)

Some acknowledged that they were developing personally, by dampening or learning to control their emotions. However, at the same time, one student also illustrated this development in their personal identity may come with a fear of losing a valued part of herself in the process.

“You get more control of your emotions and you’re able to you know, not just automatically display everything you’re feeling so I think I’m sort of, I think I’ve definitely grown up a lot and in some good ways and there are some things where I feel like I’m losing which I wish I could hang onto. Like I value my sensitive side, you know, I was an oversensitive child and so I’m glad that I’m not as sensitive as I was, but I don’t want to lose it all because I value the part of me that cares about how other people feel and if you lose all of that, what do you have left?” (Jemma, 1^st^ year of clinical medicine)

### Professional environment, roles and responsibilities

This theme looks at the working environment of the hospital, the professional role of the doctor and the responsibilities the role affords. Students experienced death as an ordinary occurrence, and many were surprised by the fact that the day just continued as usual with little acknowledgement of death:

“The people were still walking around the corridor talking, there was still dinner getting pushed up and down the hallway and stuff and the person died and nothing happened and maybe you kind of subconsciously you have an idea that there will be, that amazing things would happen at that time but nothing happened.” (John, 1^st^ year of clinical medicine)

Along the journey, students also learned about their professional role and responsibilities in processing the formalities of a death and how the hospital system works:

“We were just standing there looking at him and then someone said something like say the time of death or something like that and I was like, oh my God, that really happens you know, actually someone has to say the time of death and there’s people here who actually have to sign all that kind of paperwork and stuff and it just kind of brought me back to reality the kind of formalities that we have to do if someone dies, it’s not just as easy as die and that’s it.” (Jade, 2^nd^ year of clinical medicine)

Being involved in a patient death enabled our students to begin thinking about their professional responsibility. Despite not having any true responsibility towards a patient’s death, our students started to feel that their involvement was important and often questioned their role in the patient’s care:

“Even though I wasn’t responsible for him and I wasn’t the one taking care of him, it kind of felt like I was more, it was more my job to keep him alive so it, it was just starting to feel like it was my responsibility for him being dead” (Robyn, 1^st^ year of clinical medicine)

## Discussion

This study investigated undergraduate medical students’ experiences of death during their clinical years of training. The experiences students faced following the death of a patient reflected further insights into learning about the role of the doctor, the practice of medicine, personal identity, and the professional social organization of clinical institutions.

One new insight was the change in students’ perception about the role of the doctor and of practicing medicine following the death of a patient. This change was quite prominent and a transformative one. Here it seems that students have learned an expectation about the doctor’s role being a curative one “the aim of the game is to cheat death” and “that’s not what you expect to do as a doctor…you don’t expect your patient’s to die” but this does not match the reality of their clinical practice, and students begin to undertake a different approach “the best you can do is minimize the risk and…that’s changed my attitude in that I’m probably now less naive about what I expect to happen”*.*

It is argued that the healer aspects of medicine (e.g. attending to suffering, restoring functional capacity) are undervalued due to the dominant curing approach (elimination of the cause of the disease or reversal of the illness concerned) [15], which, as our findings show, extends to an expectation of the doctor’s role of curing your patients.

The change from a curing perspective of doctoring to a broader role of the doctor, one of caring or ‘healing’ is a necessary one. It is estimated that chronic diseases account for over half of all causes of death globally [16]. With chronic care developing as a major area for healthcare services, it is essential that medical students recognize the importance of an approach that attempts to relieve suffering, attends to restoring some functional capacity and care for those that cannot be cured.

Through experiencing the death of a patient, students have interacted with the limitations of the curative approach along with beginning to understand the necessity of the caring approach in a meaningful and powerful way. Therefore, any opportunity to embed this approach in medical students in a positive, safe, and meaningful manner is important to facilitate.

In this study, students recounted emotional distress; employed coping strategies such as socializing and talking with peers and colleagues; and struggled with balancing between becoming emotionally detached and delivering an empathic approach towards their patients. These findings corroborate previous research [7–10]. Support from staff was also highlighted by students in this study as an important and significant mechanism for coping with the death of a patient, reflecting similar findings [9, 10]. However, whether the positive support occurs frequently is questionable, with reports that emotional support from staff is not always forthcoming [9, 11].

The death of a patient also revealed further insights into the professional and social environment students will be part of in the near future along with their professional roles and responsibilities. One explanation for the students’ surprised perception of the banality, or ordinariness of death in the working environment of the hospital, we argue, is a reaction towards what Chambliss [17] refers to as routinization.

Chambliss [17] investigated the moral life of nurses in hospital settings. However, we propose his work has a lot of crossover for medical students and doctors. Chambliss [17] argues that hospitals are the same as other large organizations with one ‘crucial’ difference. In hospitals “as a normal part of the routine, people suffer and die” ([17], p.16). Acknowledging this crucial difference is important “since adapting themselves to pain and death is for hospital workers the most distinctive feature of their work.” [17, p.17].

Chambliss argues that routinization, or the acceptance of the hospital as a normal place, entails several things. These are: the continual repetition of actions, actions that break usual taboos, and that “routine is embedded in behaviour” ([17], p.24). We will look at these components in respect to medical students beginning clinical medicine.

The tasks of a healthcare worker are repeated over and over, many times a day, countless times each year. What the healthcare workers experiences, they have experienced numerous times. The event of the death of a patient is frequent (this can vary depending on what area of the hospital you work) – occurring regularly in the hospital – it becomes a routinized part of hospital life. This sense of similar events reoccurring forms a part of routinization [17]. In contrast, for students entering clinical medicine, their curriculum involves moving from attachment to attachment. What the healthcare workers experience many times, the student may be seeing for the first time, including the death of a patient – it is not routine for students.

Bodies as seen by people outside clinical institutions, are viewed as special or even sacred. However, how bodies are interacted with in the clinical setting can violate this view of bodies being special, and can be treated in a sacrilegious manner, therefore infringing on this taboo of bodies being regarded special or sacred (e.g. exposed to and touched frequently by strangers, invaded by needles). We propose that many medical students still view the body as special and/or sacred [18]. However, in a clinical institution such as the hospital, bodies are not always treated as special or sacred – which offers an explanation about why after the death of a patient the student is expecting “amazing things to happen” – but it does not. The body is treated differently, and it is evident in our results that students are initially shocked, bewildered, and are struggling to make sense of this.

Lastly, Chamberliss^19^ argues that routinization of the abnormal encompasses an entire way of performing that comprises both physical and cognitive processes. Routinization is not “all in the head” ([17], p.28). It is exhibited in the persona of a health professional going about their work while immersed in what can be perceived as abnormal events from an outsider – in this instance the death of a patient. As medical students are only just beginning their clinical lives, they have not had the time to fully acquire these embodied ways of knowing.

Hence, the life of the health professional, the insider, is commonplace and usual, as opposed to unusual [17]. The health professional’s very way of being and working in the hospital and dealing with distressing events is different from the outsider, which we argue, includes the newly arrived medical student.

### Implications

When considering educational strategies to help students when experiencing the death of a patient, literature has stressed an emphasis on targeting the clinical years [7–10]. Previous studies have emphasized student perceptions that having pre-clinical training in end-of-life (EOL) care would have limited impact on their experiences with the death of a patient in their clinical years [7, 10, 11]. A focus on positive role-modeling from clinical staff [7–11], training staff for formal or informal debriefing or support sessions [7–9, 11], mentoring led by staff [8–11], and reflective practice [10] including keeping reflective journals [8, 11] have also been advocated.

However, like Wear [11], we argue that curricula involving death and dying should be “integrated, rather than isolated in the curriculum” ([11], p.275). An integrated approach, although counter to previous studies [10, 11], we suggest, should also extend to the pre-clinical years as many learning theories emphasize prior learning as a fundamental characteristic to build upon in future learning [19]. Yet, we agree with Wear [11], that this approach needs to focus on exercises that develop personal reflection on death and dying in a variety of contexts such as historical, cultural, societal, and philosophical perspectives in order to be successful. The quote in the title of this article suggests one student was “not expecting their patient would die.” The fact that we are all mortal is a truth that must be grasped existentially, not merely intellectually [20]. Evidence suggests that addressing this in pre-clinical years does have value [21].

One of the most frequent suggestions to help students is for staff to illustrate positive role modeling via engaging with students about their experience of the death of a patient in the clinical setting. Ratanawongsa [10] calls for faculty development in EOL care to be instigated given the effect it can have on medical students. However we propose, that faculty development go further than training in EOL care, as this can lead to focusing purely on palliative care. Whereas as this study illustrates, experiencing the death of a patient can occur in many different settings, including palliative care. We suggest development in staff knowledge and practice of the hidden curriculum, and the significant influence staff have in facilitating this implicit curriculum with students to address the various settings experiencing a patient death can occur.

The hidden curriculum involves the undeclared, non-explicit messages that students pick-up from their learning environments, and as Hafferty states is a “set of influences that function at the level of the organizational structure and culture” ([22], p.404). Elements of the hidden curriculum for medical students can include the attitudes, beliefs and values espoused by the medical community. In workplace learning, these elements can be evident in many people in the working environment in which medical education is situated, and includes patients, doctors, and nurses amongst others. Therefore, the hidden curriculum has an influence on how *to be* a doctor, which includes how to cope with distressing events (e.g. a doctor should just ‘soldier on and deal with it’ or ‘a doctor should discuss distressing events with colleagues/students’), such as the death of a patient [23].

It is unknown how much attention the hidden curriculum is given in terms of staff development for clinical educators. One study indicates that the knowledge of the hidden curriculum amongst clinical staff is variable from not being aware of the term at all, to familiarity with what the hidden curriculum is and involves [24]. However, of staff that did have knowledge of the hidden curriculum, how they put their knowledge of the hidden curriculum into practice was not explored. Maybe it is time for the hidden curriculum to become more explicit and focused upon in the staff development of clinical educators that moves from *knowing about* to *working with*.

### Limitations and strengths

Our study had several limitations. Due to the method of recruitment of participants, along with a small sample size, it is not possible to make widespread generalizations around the findings of the study. Students were interviewed at different times throughout each year of their clinical education, this may have affected the recall of accuracy of the events they experienced.

Although having a medical student (HM) code the data initially may open up the analysis to certain insider bias, this was minimised by having the second coder (AB). Conversely, having a medical student analyse the data can provide valuable insights and perspectives that staff do not ‘see’.

In the study, the same person who was also medically qualified, undertook all of the interviews. He was not involved in any teaching at the medical school or clinical teaching sites. We believe this had several strengths. Using the same interviewer for all the students over the three clinical years may have helped gain trust, develop rapport, and facilitated students to feel comfortable in telling their stories. Being medically qualified may have helped in talking about both positive and negative experiences, as he was once a medical student himself.

## Conclusion

For the vast majority of students entering their clinical education they walk into a totally new and foreign environment. Yet, one area that this research identifies as important to consider and has not been examined in the literature, is for students to learn about the social organization of clinical institutions, or more specifically, the working environment of clinical institutions in regards to how death is viewed and worked with.

Focusing on learning about the social and working environment of clinical institutions is not something totally new,[25, 26] we could not find any medical education literature that discusses medical students learning about the social working environment of clinical institutions regarding how death is viewed and worked with on a daily basis. Given the reactions by students to their social working environment, this is an important area to consider so that novice clinical students can have another way of interpreting, and making sense of, their new and sometimes strange clinical environment.

The death of a patient led to some transformative learning experiences for students, from changing their approach to the practice of medicine to shaping different personal views of death. An integrated curriculum of pre-clinical and clinical learning, with an emphasis on senior staff development on the hidden curriculum is needed. Thenceforth, staff can seize the opportunity to engage with students constructively, from a variety of contexts when they experience the death of a patient. One context, that is currently missing when addressing students learning about the death of a patient, that of a social organizational perspective, seems a logical and positive move to assist medical students in learning about the profession and environment which they will be entering.

### Ethical approval and consent to participate

Ethical approval was granted for this study from the New Zealand Multi-region Ethics Committee. Informed consent for involvement in the study was obtained from the participants.

### Availability of data and materials

As data sharing was not stipulated in the approved ethics committee application, data for this paper will not be shared. We did not have consent from the participants to do so.

## Abbreviations

ELM: early learning in medicine
ALM: advanced learning in medicine
EOL: end-of-life care
